# Five-year follow-up of children with perinatal HIV-1 infection receiving early highly active antiretroviral therapy

**DOI:** 10.1186/1471-2334-9-140

**Published:** 2009-08-26

**Authors:** Elena Chiappini, Luisa Galli, Pier-Angelo Tovo, Clara Gabiano, Catiuscia Lisi, Stefania Bernardi, Alessandra Viganò, Alfredo Guarino, Carlo Giaquinto, Susanna Esposito, Raffaele Badolato, Cesare Di Bari, Raffaella Rosso, Orazio Genovese, Massimo Masi, Antonio Mazza, Maurizio de Martino

**Affiliations:** 1 Department of Pediatrics, University of Florence, Florence, Italy; 2 Department of Pediatrics, University of Turin, Turin, Italy; 3 Department of Statistics, University of Florence, Florence, Italy; 4 Pediatric Clinic, "Bambino Gesù" Hospital, Rome, Italy; 5 Infectious Diseases Unit- Department of Paediatrics, "L. Sacco" Hospital, University of Milan, Milan, Italy; 6 Department of Pediatrics, "Federico II" University, Naples, Italy; 7 Department of Pediatrics, Padua University, Padua, Italy; 8 Department of Maternal and Pediatric Sciences," Milan University, Fondazione IRCCS Ospedale Maggiore Policlinico, Mangiagali e Regina Elena, Milan, Italy; 9 Pediatric Clinic, Brescia University, Brescia, Italy; 10 Clinic of Infectious Diseases, " Giovanni XXIII" Pediatric Hospital, Bari, Italy; 11 Infectious Diseases Clinic, University of Genoa, San Martino Hospital, Genoa, Italy; 12 Pediatric Intensive Unit, Gemelli Hospital, Rome, Italy; 13 Pediatric Clinic, "S. Orsola" Hospital, Bologna University, Bologna, Italy; 14 Paediatic Unit, "S. Chiara" Hospital, Trento, Italy

## Abstract

**Background:**

Early highly active antiretroviral therapy (HAART), started within the first months of age, has been proven to be the optimal strategy to prevent immunological and clinical deterioration in perinatally HIV-infected children. Nevertheless, data about long-term follow-up of early treated children are lacking.

**Methods:**

We report data from 40 perinatally HIV-infected-children receiving early HAART, with a median follow-up period of 5.96 years (interquartile range [IQR]:4.21–7.62). Children were enrolled at birth in the Italian Register for HIV Infection in Children. Comparison with 91 infected children born in the same period, followed-up from birth, and receiving deferred treatment was also provided.

**Results:**

Nineteen children (47.5%) were still receiving their first HAART regimen at last follow-up. In the remaining children the first regimen was discontinued, after a median period of 3.77 years (IQR: 1.71–5.71) because of viral failure (8 cases), liver toxicity (1 case), structured therapy interruption (3 cases), or simplification/switch to a PI-sparing regimen (9 cases). Thirty-nine (97.5%) children showed CD4^+ ^T-lymphocyte values>25%, and undetectable viral load was reached in 31 (77.5%) children at last visit. Early treated children displayed significantly lower viral load than not-early treated children, until 6 years of age, and higher median CD4^+ ^T-lymphocyte percentages until 4 years of age. Twenty-seven (29.7%) not-early treated vs. 0/40 early treated children were in clinical category C at last follow-up (P < 0.0001).

**Conclusion:**

Our findings suggest that clinical, virologic and immunological advantages from early-HAART are long-lasting. Recommendations indicating the long-term management of early treated children are needed.

## Background

The issue of when to start highly active antiretroviral therapy (HAART) in perinatally HIV-infected children is topical [1]. Results of a clinical trial investigating the impact of early HAART on African children have been recently reported [2]. Among more than 350 children, early HAART resulted in a 76% reduction in mortality, at 10 month follow-up, confirming that this strategy might be the preferred choice with respect to deferred therapy. Nevertheless, data regarding the long-term follow-up of early treated children are lacking. Issue regarding possible side effects of HAART and lack of compliance have been arisen [1]. We previously demonstrated benefits from early HAART in perinatally infected children, on the basis of an observational study on a large cohort of Italian children prospectively followed-up from birth [3]. Hereby we update our previous findings extending the follow-up to five years.

## Methods

### Study objective

To compare clinical, immunological and virologic data of HIV-1 infected children receiving early or deferred HAART, over a median 5-year follow-up, through an observational non randomized study.

### Data collection

The Italian Register for HIV Infection in Children is a network of 106 pediatric clinics distributed throughout Italy that is highly representative of the Italian population of perinatally HIV-infected children; details are described elsewhere [4,5]. Approval for the study was granted from the University of Florence and the ethics committees of the participating institutions, and written informed consent was obtained from children's parents or legal guardians.

Infection was defined by the persistence of HIV-1 antibodies after 18 months of life or by the detection, on at least 2 occasions, of virus markers (proviral DNA, or viral RNA). Viral loads were evaluated quantitatively by Amplicor HIV Monitor test and results were expressed as Log_10 _HIV-1 RNA copies/mL. CD4^+ ^T-lymphocyte counts were measured using the standardized fluorescent-activated cell sorting technique. Based on the USA guidelines for the use of antiretroviral agents in children [6], CD4^+ ^T-lymphocyte percentages, rather than their absolute counts, were taken into account as these percentages more accurately reflect their immune status. HIV-1-related clinical events were evaluated according to the CDC definition [7].

### Treatment

The specific therapy offered was left to the discretion of single participating centres. Treatments were considered if they were given continuously for at least 1 month.

### Patients

Patients in the early treatment (n = 40) group were children with perinatal HIV-1 infection enrolled in the Register at birth, born between January 1996 and January 2007, satisfying the following conditions: a) treated with HAART (≥3 antiretroviral drugs) ≤ six months of age; b) in clinical category CDC N, A, or B before starting HAART; c) in immunological category CDC 1 or 2 before starting HAART. Children should remained free of clinical category C and/or immunological category 3 diagnoses for 1 month after initiating therapy. Additionally, data from these children were compared with those from not-early treated children born in the same period, followed-up from birth (n = 91). Children who were in CDC category C before 6 months of age have been excluded from the analyses.

### Statistical analysis

Age, CD4^+^T-lymphocyte percentages, and viral loads were expressed as median and interquartile range (IQR). When the HIV-1 RNA levels were below the lower quantification limit of the assay, a value was assigned equal to the lower quantification limit when analyzed as a quantitative variable.

Children receiving early or deferred HAART were compared by viral load, and CD4^+ ^T-lymphocyte percentages using the Mann-Whitney test. Kaplan-Meier analysis with Log rank test was performed to analyse differences in survival and survival free from CDC category C clinical events. The statistical analyses were performed using the SPSS software package (SPSS 11.5; Chicago, IL). P < 0.05 was considered statistically significant.

## Results

Early treated children were followed-up for 5.96 years (median; interquartile range [IQR]:4.34–7.48). Median age at starting HAART was 3.48 months (IQR:2.61–4.14). Nineteen (47.5%) children were still on their first HAART regimen at last follow-up. In the remaining children the first regimen was discontinued because of viral failure (8 cases), liver toxicity (1 case), structured therapy interruption (3 cases), or simplification/switch to a protease inhibitor-sparing regimen (9 cases). Thirty-nine (97.5%) children had CD4^+ ^T-lymphocyte values >25%, at last check. Undetectable viral load persisted in 31 (77.5%) children at last visit (Table 1).

**Table 1:** Characteristics of 40 perinatal HIV-infected children receiving HAART before 6 months of age.

*CDC category*	*Age at first HAART (years)*	*First HAART Regimen*	*Age at first regimen discontinuation (years)*	*Reason for discontinuation*	*Age at last follow-up (years)*	*CD4^+ ^T cells at last follow up (%)*	*Viral load at last follow-up* *(Log_10 _copies/mL)*
B1	0.21	DDI+D4T+LPV/r	6.40	Simplification	9.83	42	<1.69
A1	0.18	3TC+D4T+ LPV/r	6.65	Simplification	8.87	28	1.85
B1	0.26	AZT+3TC+NFV	Ongoing		8.67	38	<1.69
B2	0.48	AZT+3TC+NFV	Ongoing		8.66	37	3.00
A1	0.27	3TC+D4T+NFV	5.00	Simplification	8.75	45	<1.69
A2	0.08	3TC+D4T+NFV	Ongoing		8.81	35	<1.69
B1	0.30	AZT+3TC+NFV	4.47	Simplification	8.03	35	<1.69
A1	0.33	ABC+3TC+NFV	7.17	Virologic failure	7.70	28	2.63
A2	0.44	AZT+3TC+NFV	Ongoing		8.32	33	<1.69
N1	0.34	AZT+3TC+NVP	3.89	Therapy interruption	5.43	38	3.73
A2	0.34	3TC+D4T+NFV	Ongoing		6.94	37	<1.69
A1	0.33	AZT+3TC+NFV	1.50	Therapy interruption	7.41	31	5.10
A1	0.39	DDI+D4T+NFV	5.71	Simplification	7.28	32	4.00
N1	0.21	DDI+D4T+NFV	Ongoing		7.11	30	<1.69
A1	0.34	AZT+3TC+NFV	4.58	Simplification	7.78	44	<1.69
N2	0.13	3TC+D4T+NVP	Ongoing		6.40	44	<1.69
A2	0.28	AZT+3TC+NFV	Ongoing		6.08	25	<1.69
A1	0.30	3TC+D4T+NFV	6.21	Simplification	6.28	43	<1.69
A2	0.18	DDI+D4T+NFV	3.50	Virologic failure	6.03	39	<1.69
B1	0.36	3TC+D4T+NFV	Ongoing		6.24	38	<1.69
A2	0.19	DDI+D4T+NFV	1.64	Therapy interruption	6.56	20	<1.69
N1	0.25	AZT+3TC+NVP	Ongoing		5.19	42	<1.69
N1	0.42	3TC+D4T+NFV	1.71	Virologic failure	4.39	39	<1.69
A1	0.32	3TC+D4T+NFV	4.30	Simplification	5.04	37	<1.69
N1	0.27	AZT+3TC+NFV	3.21	Simplification	5.15	43	<1.69
N1	0.22	3TC+D4T+NFV	Ongoing		4.66	32	<1.69
A1	0.48	AZT+3TC+NVP	Ongoing		5.56	34	<1.69
A1	0.45	AZT+3TC+NFV	2.34	Virologic failure	5.88	39	<1.69
B2	0.15	ABC+D4T+NVP	Ongoing		4.18	32	<1.69
B1	0.45	3TC+D4T+NFV	2.78	Virologic failure	5.06	28	2.34
B2	0.22	3TC+D4T+ LPV/r	3.77	Virologic failure	5.10	38	<1.69
A2	0.14	AZT+3TC+NVP	Ongoing		2.02	51	<1.69
B1	0.43	AZT+3TC+NVP	Ongoing		3.89	36	<1.69
N1	0.31	3TC+D4T+NFV	Ongoing		2.27	35	2.50
B1	0.24	3TC+D4T+NFV	1.69	Virologic failure	3.83	32	<1.69
N1	0.31	AZT+3TC+NFV	Ongoing		0.59	33	2.96
N2	0.25	AZT+3TC+NFV	2.92	Virologic failure	3.41	33	<1.69
N1	0.17	AZT+3TC+NFV	Ongoing		2.18	35	<1.69
B1	0.36	ABC+3TC+LPV/r	0.45	Liver toxicity	2.10	42	<1.69
A1	0.23	3TC+D4T+NFV	Ongoing		0.88	35	<1.69

Note: ZDV, zidovudine; 3TC, lamivudine; D4T, stavudine; DDI, didanosine; NELF, nelfinavir; LPV/r, lopinavir/ritonavir; NEV, nevirapine; ABC: abacavir.

Data from early treated children were compared with data from 91 children not receiving early therapy born in the same period and followed-up from birth (median follow-up: 6.9 years [IQR:2.8–9.76]; p = 0.218 *vs. *early treated children). Median age at first HAART regimen was of 2.21 years (IQR:1.37-4-76). Eighteen children (19.78%) were in CDC clinical category N; 19 (20.9%) in A; 27 (29.7%) in B, and 27 (29.7%) in C categories, at last observation.

Early treated children displayed significantly lower viral load than not-early treated children until 6 years of age (Table 2). Higher median CD4^+ ^T-lymphocyte percentage was evident until 4 years of age but not at higher age classes (Table 3). A last follow-up visit 50/91 (54.9%) not-early treated *vs. *31/40 (77.5%) early treated children reached undetectable viral load (P = 0.001).

**Table 2:** Differences in viral loads among 40 early treated children and 91 children receiving deferred treatment, by age

Age (years)											
		<1	1	2	3	4	5	6	7	8	9

Median Viral load (Log RNA copies/mL) (IQR)	Early treated children	5.6 (3.3–5.7)	2.6 (1.7–3.8)	1.7 (1.7–3.0)	1.7 (1.7–2.6)	1.7 (2.6–3.5)	1,7 (1.7–2.8)	1,6 (1.7–2.6)	1.7 (1.9–4.5)	1.7 (1.7–2.1)	1.7 (1.7–1.7)
	
	Not-early treated children	5.2 (3.9–5.7)	4.4 (3.6–5.2)	4.0 (2.6–4.7)	3.6 (1.8–4.6)	3.6 (1.7–4.2)	3.0 (1.7–4.2)	2.7 (1.7–3.7)	2.4 (1.7–3.6)	1.8 (1.7–3.5)	2.1 (1.6–4.0)
	
	P*	0.854	<0.0001	<0.0001	<0.0001	0.020	0.021	0.024	0.881	0.131	0.450

Note: IQR: interquartile range; *by Mann-Whitney test.

**Table 3:** Differences in CD4^+ ^T-lymphocyte percentages among 40 early treated children and 91 children receiving deferred treatment, by age

Age (years)											
		<1	1	2	3	4	5	6	7	8	9

Median CD4^+ ^T-lymphocyte percentage (IQR)	Early treated children	36 (28–50)	38 (35–43)	38 (35–42)	36 (28–40)	35 (32–41)	35 (31–42)	36 (28–39)	38 (31–39)	37 (32–38)	35 (28–42)
	
	Not-early treated children	34 (26–45)	31(23–43)	32 (24–39)	33 (27–40)	30 (22–37)	32 (25–38)	33 (35–37)	32 (26–37)	32 (27–37)	33 (24–37)
	
	P*	0.126	0.003	0.001	0.021	0.015	0.107	0.116	0.090	0.126	0.647

Note: IQR: interquartile range; *by Mann-Whitney test.

**Figure 1:**
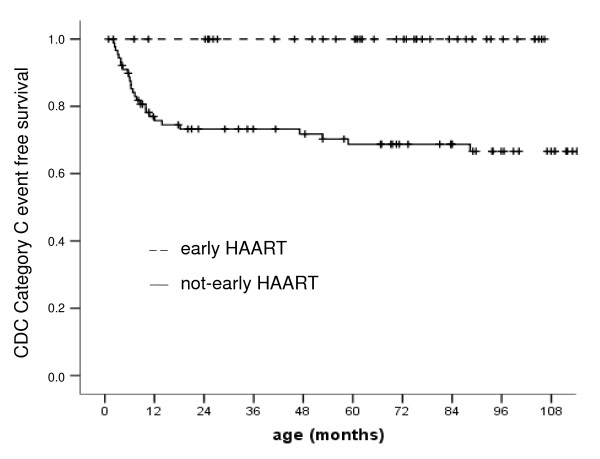
**CDC Category C event-free survival in children receiving early (- - - dotted line) and not-early HAART (--- solid line)**. early HAART 40(0) 38 (0) 36(0) 35(0) 33(0) 31(0) 20(0) 14(0) 8(0) 1(0) *Patient number (clinical event number)*. not-early HAART 91(20) 62 (3) 55 (0) 51(1) 49 (2) 44 (0) 38 (0) 34(1) 27(0) 22(0) *Patient number (clinical event number)*.

Among children receiving early therapy only one child developed a CDC clinical category A event (lymphadenopathy larger than 0.5 cm at more than two sites, at age 19 months) and no child died. Conversely, 48 (51.74%) children receiving deferred HAART showed a decline in the CDC category (9 patients falling to category A, 20 to category B, and 18 to category C) and one of these children died (at age 8.1 years, subsequently to severe sepsis complications.) (P = 0.544, by Kaplan-Meier analysis with Log-rank test for survival). Twenty-seven (29.67%) not-early treated *vs. *0/40 early treated children were in CDC clinical category C at last follow-up (P < 0.0001, by Kaplan-Meier analysis with Log-rank test) (Figure 1). Time to failure of the first HAART in children receiving deferred therapy was 34.2 months (median; IQR:28.0–45.2) in children receiving early HAART and 29.5 months (median; IQR:6.8–61.3) in children receiving deferred HAART.

## Discussion

Our study on 40 children with perinatal infection, receiving early HAART and followed up for a median of 5.96 years, demonstrated that clinical, immunological and virologic benefits persist over time, indicating that advantages from this kind of strategy are long-lasting. The fact that the difference in CD4^+ ^T-lymphocyte percentage was no more statistically significant in children older that 4 years of age may be due to the limited study number. Indeed, a trend toward higher CD4^+ ^T-lymphocyte percentage in early-treated children was evident until 9 years of age and, likely, it could be confirmed in larger studies.

With respect to our previous findings [3], we found a lower, but still considerable, proportion of early-treated children with sustained undetectable viral load at last check (77%). This rate is similar, or higher than those reported by other authors [8,9]. This finding is encouraging, even though the possibility to switch to another HAART regimen in the event of virologic failure, not only the efficacy of the initial therapy, may have influenced our results.

Also, with respect to our previous study, we investigated how children receiving early HAART have been managed over time. This is a crucial point. Indeed, even if updated guidelines consider now early therapy as a reasonable option in children [6], it is still unclear if it should be continued life-long or stopped after one or more years of age. In our study the strategy chosen by the Italian pediatricians was not-univocal. The majority of early treated children was still on their first HAART regimen at the last follow-up and displayed good immunological, virologic, and clinical parameters. Some children experienced multiple HAART regimens in their life, because of virologic failure or side effects to therapy, but, even in this group of children, no immunological or clinical deterioration was evident at last follow-up. Therapy interruption was attempted in three children. Two of them were out of therapy at last follow-up visit, maintaining good immunological and clinical parameters, while HAART reintroduction was necessary in the third child because of immunological decay. Switch to a PI-sparing regimen (mainly with abacavir or efavirenz) was the chosen option for a minority of children and it was associated with immunological, virologic and clinical success in most of them. This point has been addressed in previous studies, suggesting that this may be a good strategy [10-13].

No difference in viral load or CD4^+ ^T-lymphocyte percentage was present between the two groups at age > 1 year. Also in both groups, children who were in CDC category C before 6 months of age have been excluded from the analysis. Thus, the two groups of children should be comparable. Nevertheless, our observational study has some limitations. Potentially, unmeasured confounders may have caused the observed differences. Detailed information regarding compliance to therapy and viral resistance mutations was not available. Early treated children could have motivated families, resulting in better adherence and better outcomes. Data regarding detailed reasons that led to the treatment interruption or PI substitution were not available. Physicians who took care for these children may have simplified the therapy in order to reduce possible long-term metabolic toxicity effects of PIs. Accordingly, other authors demonstrated that a PI-sparing regimen may be a good strategy in children [10-13].

## Conclusion

In conclusion, our previous findings are substantially strengthened by these additional data on a long term follow-up, confirming benefits of early HAART. Our observational study may not solve the issue regarding the optimal strategy for long term managing of early treated children. Even if this is only a speculation, it is possible that use of a PI-sparing regimen might represent a good option. This is in agreement with results from a Belgian study, including about twenty children receiving early therapy with a protease inhibitor-sparing HAART [10]. Guidelines indicating the subsequent management of early treated children are needed.

## Abbreviations

CDC: Centers for Disease Control and Prevention; IQR: interquartile range; HAART: active antiretroviral therapy; PI: protease inhibitor.

## Competing interests

CG has been a consultant to GlaxoSmithKline, Abbott, Bristol-Myers Squibb, Tibotec, Boehringer-Ingelheim, Gilead Sciences, Pfizer, Sanofi Pasteur, and GSK-Bio, and has received research grants from GlaxoSmithKline, Abbott, Bristol-Myers Squibb, Boehringer-Ingelheim, Gilead Sciences, and Sanofi Pasteur. All other authors declare that they have no conflict of interests.

## Authors' contributions

EC, Mdm, PAT, LG and CG initiated the study, the design, and the data collection. EC and CL performed the statistical analysis and were involved in the interpretation of the results. EC and Mdm, wrote the manuscript and PAT, LG and CG contributed to the conception and design of the study and revision of the manuscript. All authors provided individual patient data, read and approved the final manuscript.

## Pre-publication history

The pre-publication history for this paper can be accessed here:

http://www.biomedcentral.com/1471-2334/9/140/prepub
